# Salivary Characteristics and Other Risk Factors Associated with the Severity of Chemical and Mechanical Tooth Wear in At-Risk Groups: A Cross-Sectional Study

**DOI:** 10.3390/jcm14207260

**Published:** 2025-10-14

**Authors:** Ona Rius-Bonet, Eva Willaert, Susana Jiménez-Murcia, Guillem Diego-Esteve, Cristina Artero, Isabel Sánchez, Isabel Baenas, María del Carmen Peña-Cala, Fernando Fernández-Aranda, Jordi Martinez-Gomis

**Affiliations:** 1 Doctorate in Medicine and Translational Research Program, Faculty of Medicine and Health Sciences, University of Barcelona (UB), L’Hospitalet de Llobregat, 08907 Barcelona, Spain; orius@ub.edu; 2 Department of Odontostomatology, Faculty of Medicine and Health Sciences (Dentistry), University of Barcelona (UB), L’Hospitalet de Llobregat, 08907 Barcelona, Spain; jmartinezgomis@ub.edu; 3 Oral Health and Masticatory System Group, Bellvitge Biomedical Research Institute (IDIBELL), 08908 Barcelona, Spain; 4 Clinical Psychology Department, Bellvitge University Hospital, 08907 Barcelona, Spain; susanajimenez@ub.edu (S.J.-M.); gdiego@idibell.cat (G.D.-E.); crarterm7@alumnes.ub.edu (C.A.); isasanchez@bellvitgehospital.cat (I.S.); ibaenas@idibell.cat (I.B.); ffernandez@idibell.cat (F.F.-A.); 5 Psychoneurobiology of Eating and Addictive Behaviors Group, Neuroscience Program, Bellvitge Biomedical Research Institute (IDIBELL), 08908 Barcelona, Spain; 6 CIBER Physiopathology of Obesity and Nutrition (CIBERobn), Instituto Salud Carlos III, 08907 Barcelona, Spain; 7 Department of Clinical Sciences, School of Medicine and Health Sciences, University of Barcelona (UB), 08907 Barcelona, Spain; 8 Department of Gastroenterology, Bellvitge University Hospital, Hospitalet de Llobregat, 08907 Barcelona, Spain

**Keywords:** tooth wear, saliva, risk factors, tooth erosion, tooth attrition, hydrogen-ion-concentration, bruxism

## Abstract

**Background/Objectives**: Tooth wear (TW) is a prevalent multifactorial condition resulting from chemical erosion and mechanical forces, yet the contributions of risk-group status and salivary factors remain insufficiently characterized. This study aimed to investigate the relationship between salivary characteristics and the severity of chemical and mechanical TW in at-risk groups, including gastroesophageal reflux disease (GERD), sleep bruxism (SB), eating disorders (EDs) and gambling disorder (GD). **Methods**: This cross-sectional observational study enrolled adults categorized into the four mutually exclusive at-risk groups and an age and sex-matched healthy control group. Demographic information, medical history, oral hygiene, diet, stress, and parafunctional habits were obtained through questionnaires. TW was assessed by a single examiner using TWES 2.0. Maximum bilateral force and salivary pH, flow and buffer capacity was measured. Correlations, multivariate linear regression, and mediation models were used to explore the relationships between the different variables and TW. **Results**: In total, 147 participants, divided into five groups (34 with GERD, 28 with SB 20 with GD, 20 with ED, and 45 controls) were included. The lowest resting salivary pH was observed in the GERD and ED groups (GERD: 6.63 ± 0.61; ED: 6.62 ± 0.52). The GERD group also exhibited the highest chemical (1.51 ± 0.58) and mechanical (1.08 ± 0.58) TW. Chemical and mechanical wear were strongly correlated, and mechanical wear increased with age. Risk-group status and salivary pH explained 47% of chemical wear, while age and bite force explained 54% of mechanical wear. **Conclusions**: Chemical TW was strongly linked to risk-group status—particularly GERD/ED—and low salivary pH, while mechanical TW related to age and bite force. Further longitudinal studies with larger samples, employing standardized methodologies and criteria are needed.

## 1. Introduction

Tooth wear (TW) refers to the loss of superficial dental tissue due to chemical (erosion) and/or mechanical processes (attrition and abrasion) that are not related to dental caries, trauma, or developmental disorders [1]. The prevalence of TW ranges from 30% to 50% in primary dentition and between 20% and 45% in permanent dentition [2].

Chemical wear may be caused by stomach acids and by exogenous acids from acidic foods and beverages [3,4]. Populations affected by gastroesophageal reflux disease (GERD) and individuals with eating disorders (EDs), particularly those who engage in self-induced vomiting, exhibit not only a higher prevalence of chemical TW, but also more severe TW [5,6,7,8,9]. Intrinsic mechanical wear results from grinding between upper and lower teeth, as observed in sleep bruxism (SB) or awake bruxism (AB) [10], whereas extrinsic mechanical wear is mainly associated with aggressive tooth brushing [11]. Notably, the most recent meta-analysis reports a global bruxism (sleep and awake combined) prevalence of 22.22%, with sleep bruxism at 21% and awake bruxism at 23%, underscoring the magnitude of the problem [12]. Therefore, several risk factors for TW have been identified, including gastroesophageal reflux disease, EDs, specific diets, acidic beverages, alcohol consumption, other legal and illegal substances, medications particularly, oral dryness-inducing agents and inhaled corticosteroids for asthma management, as well as occupational and sports-related factors [13,14]. Individuals affected by gambling disorder (GD) and other addictive behaviors may experience poorer sleep quality, higher stress levels, and increased anxiety [15]. Since these psychological states are strongly associated with AB and, to a lesser extent, SB, persons with GD, especially those experiencing higher addiction-related distress, may be more prone to jaw clenching or teeth grinding during wakefulness. However, whether individuals with GD represent risk group of TW is not well established, since no study has assessed the severity of mechanical or chemical TW of this population [13].

In the general population, several risk factors have been associated with TW, and those assessed by questionnaires or interview are categorized into several domains, including sociodemographic/socioeconomic factors, medical history, drinking habits, eating habits, oral hygiene habits, dental factors, bruxism and temporomandibular disorder (TMD), behavioral or leisure-related factors, and stress [16,17]. Age, male gender, regurgitation, and dietary factors seem to be the key factors for chemical TW, whereas oral hygiene habits and bruxism are the main factors for mechanical TW [16,17,18,19]. The reason why men have more TW are not yet understood, but it has been hypothesized that men consume more acidic drinks resulting in more chemical TW [20,21]. In addition, men have higher bite force, leading to more occlusal contact area and mechanical TW [20,22,23]. On the other hand, saliva is recognized as one of the most important protective factors against dental erosion due to its buffering capacity, remineralization potential, and role in the formation of the acquired enamel pellicle [24,25]. In conditions with frequent intrinsic acid exposure, altered salivary parameters have been described with heterogenous results. One recent systematic review and meta-analysis found that patients with gastroesophageal reflux disease exhibit significantly lower salivary pH, reduced stimulated flow rates, and diminished buffering capacity compared with healthy controls [26]. Similarly, another systematic review stated that individuals with eating disorders—especially those with bulimia—often experience decreased unstimulated salivary flow, lower pH, impaired buffering ability, and frequent xerostomia, with severity worsening alongside longer illness duration and concurrent medication use [8]. It remains unclear how stimulated and unstimulated salivary flow, pH, and buffering capacity contribute to protection against TW in individuals exposed to acid due to GERD or self-induced vomiting, as well as in individuals with either SB or AB [21,25,27].

The relationship between different salivary characteristics and the presence of TW has been studied recently in different populations, from a general population of young adults [28,29] to patients with moderate or severe TW [18,21]. However, it is still unknown what the role of salivary characteristics is in the severity of mechanical or chemical TW in individuals of different at-risk populations. Since several risk factors have correlated each other, several statistical approaches have been recommended to provide a stronger basis for causal inference, such as multivariable regression models, quantitative assessment of systematic bias, and conditional process analysis [30,31,32].

This study aimed to investigate the relationship between salivary characteristics and the severity of chemical and mechanical TW across different at-risk groups, including GERD, SB and mental disorders such as EDs and GD. Additionally, it also aimed to identify the predictors more strongly associated with the severity of chemical and mechanical TW. The null hypothesis was that there is no relationship between any salivary characteristic and the degree of TW.

## 2. Materials and Methods

### 2.1. Design and Population

A cross-sectional observational study was performed between May 2021 and January 2024 at the Dental Hospital of the University of Barcelona and at the Departments of Clinical Psychology and Gastroenterology Departments at the Bellvitge University Hospital (Barcelona, Spain). Sample size calculation was performed assuming a two-sided α = 0.05 and 80% power, indicating that 26 participants per group were required to detect a minimum between-group difference of 2.4 mL/5 min, allowing for a 5% drop-out. A common standard deviation of 2.5 mL/5 min was assumed from prior studies with comparable methodology [33,34]. Inclusion criteria for all participants were to be older than 18 years old and to have at least 10 natural teeth and a minimum of 20 functional (natural and prosthetic) teeth.

Inclusion criteria for each group were defined as follows. For the GERD group, participants were required to have experienced pyrosis and/or regurgitation within the past year and to have had a positive pH-metry result, defined as acid exposure time greater than 6%, within the previous five years. The ED group included individuals who reported self-induced vomiting for more than one year with an average frequency of at least once per week during the past three months. All individuals in this group fulfilled DSM-5 criteria (APA, 2013) [35] for purging (diagnosis of anorexia nervosa -restrictive or binge-purging subtypes-, bulimia nervosa, or other specified feeding and eating disorders-purging subtype) confirmed through a semi-structured face-to-face interview. The bruxism group consisted of participants who were aware of clenching or grinding their teeth during sleep and exhibited at least one clinical sign, such as wear facets, tongue or lip indentations, or jaw symptoms upon awakening [36]. The GD group included individuals who reported clenching or grinding their teeth while awake and who met DSM-5 criteria for GD (APA, 2013) [35]. Each risk group was defined by specific inclusion criteria, such that the eligibility requirements for each group were mutually exclusive. Finally, the control group consisted of healthy individuals who did not meet any of the specific inclusion criteria described for each risk group and were matched by age and sex.

All participants gave informed signed consent approved by two local ethics committees (Ref 37/2020 Barcelona University Dental Hospital, and Code PR127/21 Bellvitge University Hospital). All procedures were performed according to the principles of the Helsinki Declaration, and this report has been completed in accordance with the OHStat guidelines [37].

### 2.2. Clinical Procedures

A single experienced examiner who has served as the calibrated examiner in previous studies on dental wear [29,38,39] (O.R.-B.) conducted the interview to collect data on sex, age, height, weight, general health (e.g., chronic medication), general habits (e.g., consuming alcohol, tobacco or illegal drugs, regular practice of physical activity or swimming), oral hygiene, and dietary habits. Toothbrushing frequency was recorded as the number of brushings per day. The force of toothbrushing was assessed using a 10-point ordinal scale, and the type and hardness of the toothbrush was evaluated as manual/powered and soft/medium/hard. The frequency of dietary acid intake was assessed using a five-point ordinal scale and asking the participants “How many times per week do you eat/drink fruit/carbonated-beverages/fruit-juices/vinegar?”. A new variable named “acid diet” was calculated as the sum of these four scores (range, 0–16) [29,40]. Participants were also asked to complete the Perceived Stress Scale (PSS) and the Oral Behavior Checklist (OBC) and the sum score for each instrument were calculated [41,42]. The first OBC item “clench or grind teeth when asleep” represented the frequency of self-report SB. The sum of the third and fourth items “grind teeth together during waking hours” and “clench teeth together during waking hours” represented the frequency of self-report of AB.

The same researcher (O.R.-B.) performed the TW assessment according to the ‘Tooth Wear Status’ of the Tooth Wear Evaluation System (TWES 2.0) [43]. Using the five-point ordinal scale, the degree of wear on each tooth surface (occlusal/incisal, vestibular/buccal and oral/lingual) was graded in two ways, one considering chemical TW according to its clinical signs (wear on non-occlusal surfaces; flattening of convex areas; cupping, cratering, or grooving of cusps; preservation of the enamel cuff; or a dull appearance), and the other grading considered mechanical TW according to its clinical signs (plane facets in the occlusal/incisal surface that matched a plane facet in an antagonistic tooth; wear lesions in the cervical areas of canines and/or premolars) [39]. Severity of chemical and mechanical TW was determined by calculating the sum score of each grading, a value between 0 and 384 (4 score * 3 surfaces * 32 teeth). To compensate for the number of teeth scored, these values were divided by the number of natural teeth and named as severity of chemical or mechanical TW per tooth, which ranged from 0 to 12 (4 score * 3 surfaces).

Bilateral maximum bite force was measured using the Innobyte system (Kube Innovations, Montreal, QC, Canada), according to the manufacturer’s instructions. Three measurements were performed with the participant seated, and the average of the highest two values was used for analysis [44].

All salivary tests were performed between 09:00 h and 13:00 h to reduce biological and circadian variation. Participants were instructed to abstain from food, beverages, smoking, and tooth brushing for 60 min prior to sample collection to minimize contamination and salivary flow alterations. Unstimulated saliva was collected using the spitting method, with participants expectorating at 60 s intervals or upon accumulation over a 5 min period. Using the Saliva-Check Buffer system (GC Europe N.V., Leuven, Belgium) according to the manufacturer’s instructions, resting salivary consistency was evaluated visually and assessed as having normal or increased viscosity. Salivary pH was measured by placing a pH test strip into an unstimulated saliva sample for 10 s, and the result was classified as normal (pH 6.8–7.8) or acidic (pH < 6.8). Participants were instructed to chew a piece of wax for 30 s to stimulate salivation and the saliva was collected into a cup for 5 min. The stimulated flow rate was categorized as normal (≥5 mL/5 min) or reduced (<5 mL/5 min). These cut-offs were chosen according to the manufacturer’s instructions. One drop of saliva was placed onto each of three test pads and a change of color indicated the buffering ability of the saliva. The buffer capacity was categorized as normal (10 to 12) or low (0 to 9). Chair-side saliva testing kits provide a rapid, convenient, and reliably validated method for assessing salivary function in routine screenings and patient monitoring [45].

### 2.3. Statistical Analysis

Statistical analyses were conducted using R software, version 4.3.3 [46]. Participant characteristics were summarized as percentages for categorical variables and as means ± standard deviations (SD) and medians for continuous variables. Comparisons between study groups were performed using the Mann–Whitney U test or the Kruskal–Wallis test for continuous variables, and the Chi-squared test or Fisher’s exact test for categorical variables, as appropriate. Post hoc comparisons were adjusted for multiple testing using the Holm method, to control the family-wise error rate. Additionally, effect sizes for mean and proportion comparisons were assessed using Cohen’s d and Cohen’s h, with values between 0.3 and 0.7 indicative of a moderate effect, and above 0.7 of a large effect, as reported in the dental literature [47].

Bivariate relationships between study variables and chemical or mechanical severity of TW per tooth were evaluated using the Spearman correlation and graphical visualization when non-linear trends were suspected for confirmation.

To meet the normality assumption required for subsequent linear multivariate analyses, a square root transformation was applied to the severity scores of chemical and mechanical TW per tooth. The normality of transformed variables was verified using the Shapiro–Wilk test within each risk group. Additionally, when the ‘Group’ variable was included in the model, the Control group was consistently used as the reference category.

Two multiple linear regression models were constructed using a bidirectional stepwise selection procedure (forward and backward) to examine whether variables were significantly associated with the chemical or mechanical severity of TW per tooth. Every potential variable was considered, and the selection process was iterated until convergence, with the Akaike Information Criterion (AIC) employed as the optimization parameter to balance model fit and complexity and reduce the risk of overfitting, always providing clinical interpretation [48]. All underlying assumptions of the regression model were evaluated.

Finally, mediation analyses were conducted to explore associations between variables within each tooth wear subtype. First, a simple model was used to assess the role of salivary pH as mediator between risk group membership and severity of chemical TW per tooth, adjusted by sex and age. Secondly, another mediation model was conducted to evaluate whether bite force mediated the relationship between age and mechanical TW severity per tooth, adjusted for sex.

## 3. Results

### 3.1. Incidences

Out of the 200 individuals invited to participate, 3 did not meet the inclusion criteria, 11 declined participation, 21 were unavailable for scheduling, and 18 failed to attend their scheduled appointment. Additionally, one participant did not complete the salivary test, and bite force measurements were missing for 63 participants. As a result, data from 147 participants were included in the final analysis, acknowledging partial missingness in specific variables as noted above.

### 3.2. Description of the Population

Table 1 and Table 2 present the characteristics of the participants by group. Although age and sex distributions varied across some groups, the control group was balanced with the risk group in terms of age and sex.

**Figure 1:**
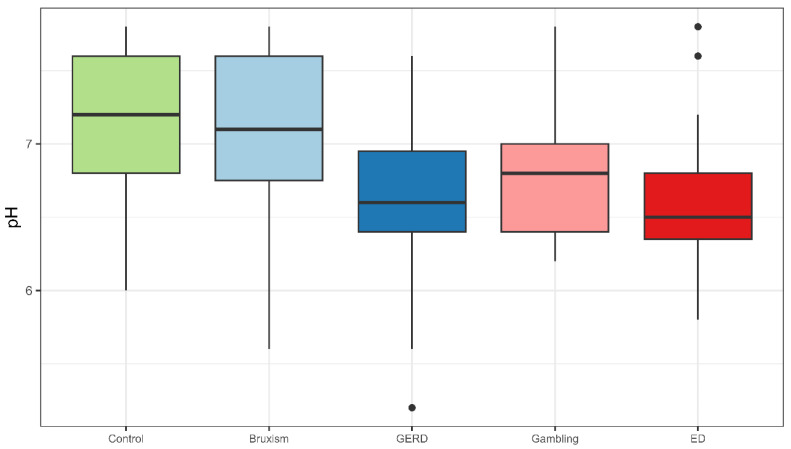
Salivary pH distribution across groups. Abbreviations: GERD (Gastroesophageal Reflux Disease), ED (Eating Disorder).

Salivary pH was significantly more acidic in the GERD and ED groups compared to the sleep bruxism, gambling, and control groups (*p* < 0.05; Kruskal–Wallis test) (Figure 1). Participants with GERD also exhibited lower salivary flow rates compared to the control group, while no significant differences in salivary buffer capacity were observed among the groups.

**Figure 2:**
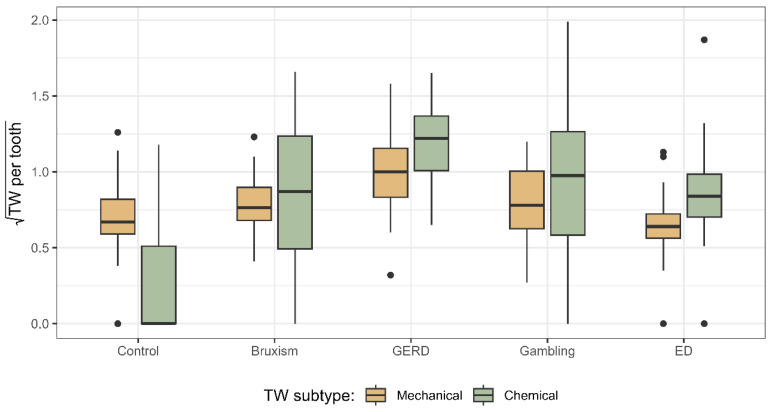
Chemical and mechanical TW distribution across groups. Abbreviations: GERD (Gastroesophageal Reflux Disease), ED (Eating Disorder), TW (Tooth Wear).

Participants in the GERD group exhibited the highest severity of both chemical and mechanical TW per tooth (Figure 2). Although individuals in the control group had the lowest values of chemical TW per tooth, their mechanical TW severity was comparable to that observed in the gambling, ED, and sleep bruxism groups (*p* > 0.05; Kruskal–Wallis test).

### 3.3. Bivariate Relationships

**Figure 3:**
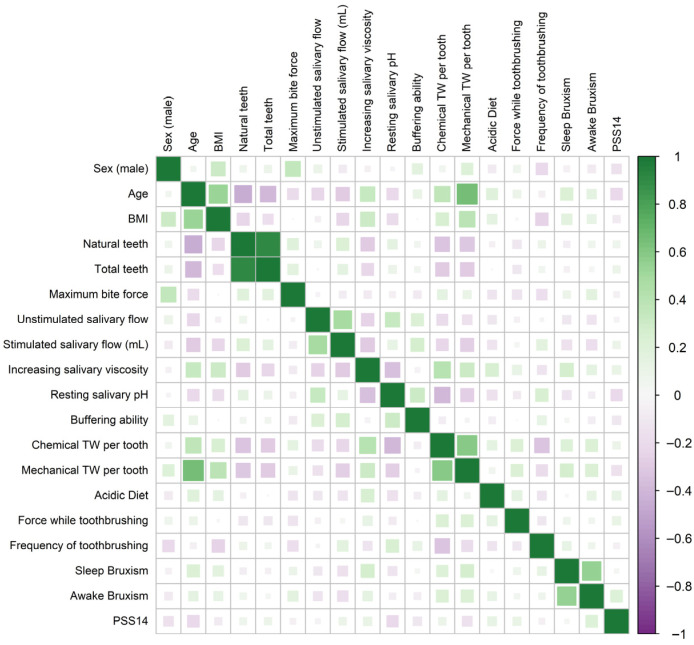
Spearman’s correlation matrix of the main study variables. Abbreviations: BMI (Body Mass Index), TW (Tooth Wear), PSS (Perceived Stress Scale).

**Figure 4:**
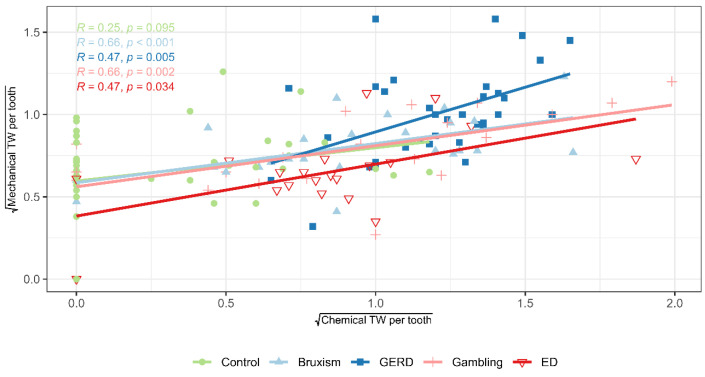
Relationship between chemical and mechanical tooth wear across study groups. Scatterplot, linear regression lines and correlation coefficients. Abbreviations: GERD (Gastroesophageal Reflux Disease), ED (Eating Disorder), TW (Tooth Wear).

**Figure 5:**
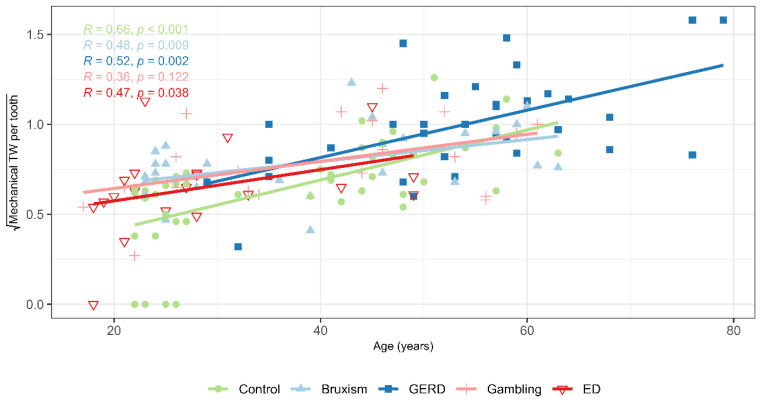
Relationship between years of age and mechanical tooth wear across study groups. Scatterplot, linear regression lines and correlation coefficients. Abbreviations: GERD (Gastroesophageal Reflux Disease), ED (Eating Disorder), TW (Tooth Wear).

The correlation matrix (Figure 3) and scatter plot (Figure 4) revealed a strong association between the severity of chemical and mechanical TW per tooth (Spearman’s ρ = 0.598, *p* < 0.001), particularly within the risk groups. The strength of this correlation ranged from ρ = 0.47 in the GERD group to ρ = 0.66 in the sleep bruxism group. A second strong correlation was observed between age and the severity of mechanical TW per tooth (Spearman’s ρ = 0.640, *p* < 0.001), most pronounced in the control group. Among the risk groups, the correlation between age and mechanical TW ranged from ρ = 0.36 in the gambling group to ρ = 0.52 in the SB group (Figure 5).

The severity of chemical TW per tooth was significantly associated with age, number of teeth, and body mass index (BMI). Correlation matrix also revealed associations between the severity of mechanical TW per tooth and several variables: age, sex, number of teeth, frequency and force of toothbrushing, as well as both SB and AB. Age was positively correlated with BMI, acid dietary intake, SB, salivary dehydration, and salivary viscosity, and negatively correlated with the number of natural teeth, perceived emotional stress, salivary pH, and both stimulated and unstimulated salivary flow rates.

Male participants exhibited higher BMI and bite force compared to females. Additionally, they showed greater mechanical TW, reported lower toothbrushing frequency, and had fewer oral habits. Salivary dehydration and buffer capacity levels were also reduced in males.

Several salivary characteristics were intercorrelated. The strongest correlations were observed between salivary viscosity, salivary pH, and salivary flow rate, as well as between buffer capacity, salivary pH, and salivary flow rate. However, salivary viscosity was not correlated with buffer capacity.

### 3.4. Multivariate Relationships

Multiple regression analysis identified at-risk groups (GERD, SB, GD, or ED) and salivary pH as the variables most strongly associated with the severity of chemical TW per tooth (Table 3). Together, these two predictors explained 46.9% of the variance in the outcome (adjusted *R*^2^ = 0.45; *p* < 0.001).

Forward multiple regression analysis revealed that age, followed by maximum bite force, were the variables most strongly associated with the severity of mechanical TW per tooth (Table 4). These two predictors explained 53.8% of the variance in the outcome (adjusted *R*^2^ = 0.527; *p* < 0.001).

**Figure 6:**
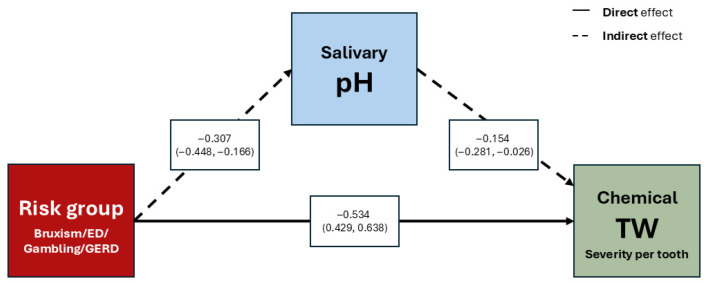
Mediation analysis showing the effect of risk group on chemical tooth wear severity per tooth, mediated by salivary pH. Normal line represents direct effect of risk group. Dotted line represents the mediated effect. Estimates are adjusted for sex and age. Abbreviations: GERD (Gastroesophageal Reflux Disease), ED (Eating Disorder), TW (Tooth Wear).

Mediation analysis, adjusted by age and sex, demonstrated that belonging to any risk group exerts a significant positive direct effect on the severity of chemical TW (*p* < 0.001), as well as a significant positive indirect effect mediated by salivary pH (*p* = 0.043) (Figure 6) (Table 5).

**Figure 7:**
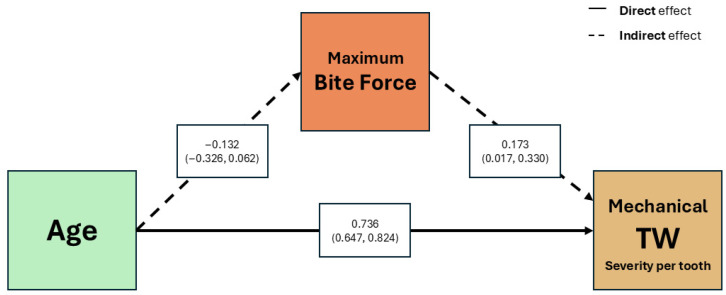
Mediation analysis showing the effect of Age on mechanical tooth wear severity per tooth, mediated by Maximum bite force. Normal line represents direct effect of Age. Dotted line represents the mediated effect. Estimates are adjusted for sex. Abbreviations: TW (Tooth Wear).

According to the mediation model constructed to assess the role of maximum bite force as a mediator between age and the severity of mechanical TW, adjusted by sex, both age and maximum bite force were positively associated with the severity of mechanical TW per tooth, but the negative indirect effect of age to maximum bite force was not significant (Figure 7). The standardized total effect of age on severity of mechanical TW per tooth was 0.713 (Table 6).

## 4. Discussion

This study shows that belonging to an at-risk group and exhibiting lower salivary pH are the variables most strongly associated with chemical TW. Accordingly, the null hypothesis of no relationship between salivary characteristics and TW is partially rejected, as salivary pH showed a significant association with chemical TW, whereas salivary flow rate and buffer capacity did not.

Both GERD and ED risk groups exhibited lower salivary pH, and GERD patients also showed the lowest stimulated salivary flow rate, while buffering capacity did not differ significantly among groups. These findings align with previous research linking intrinsic acid exposure to altered salivary parameters, although results remain inconsistent due to methodological limitations [26,27,49]. The qualitative assessment of the systematic review by Madariaga et al. [25] reported an inverse correlation between salivary pH and flow and TW, but found no clear association with buffering capacity, likely due to limitations of colorimetric strip methods. Similarly, a meta-analysis restricted to patients with GERD concluded that salivary pH, resting flow, and buffering capacity may be reduced, although evidence remains uncertain due to variability in GERD diagnosis and salivary testing protocols [26].

GERD is a well-known risk factor for TW, reflecting alterations in salivary protective function. Moazzez et al. [50] reported reduced buffering capacity and lower stimulated salivary flow in patients with hoarseness. Similarly, Sujatha et al. [51] and Bechir et al. [52] found decreased salivary pH, flow, and buffering capacity associated with TW. Proton pump inhibitor use, and chronic acid exposure may further impair salivary gland function [26].

Similarly, patients with EDs and self-induced vomiting experience recurrent acid exposure and salivary dysfunction [6,53]. Evidence on salivary alterations in EDs shows heterogeneous results. While some studies reported lower oral pH [54] or reduced unstimulated salivary flow associated with greater erosion severity [55], others identified decreased pH, bicarbonate, and phosphate levels leading to reduced buffering capacity [56]. A systematic review confirmed that, despite methodological differences, reduced salivary pH and flow are frequent findings and represent key risk factors for TW in these patients [53]. In the present study, however, no significant differences in salivary flow or buffering capacity were observed in this group, possibly due to the lower chronicity of ED and the small sample size [25].

Regarding the SB group, salivary parameters were within normal ranges, aligning with previous findings [18,29,57]. These results might suggest that saliva plays a limited role in mechanical wear under normal conditions but may become relevant in patients with comorbidities or reduced salivary flow, which could contribute to TW by decreasing oral lubrication [10,58].

Interestingly, the group with GD also exhibited significantly lower salivary pH despite normal flow and buffering capacity. This is the first report on salivary parameters and TW in GD, a population characterized by stress dysregulation, psychiatric comorbidities, all known to affect salivary composition [59,60,61].

In terms of TW severity, notable differences were observed depending on the underlying aetiology. Patients with GERD presented the greatest severity of both chemical and mechanical wear, suggesting a synergistic effect between acid erosion and attrition, as acid-mediated demineralization increases susceptibility of dental surfaces to forces from mastication, bruxism, and parafunctional habits [10,62,63]. GERD is frequently associated with SB, conferring a 4.7-fold increased risk of severe TW [63]. Additionally, GERD-associated salivary dysfunction and older age may contribute to greater loss [64].

By contrast, ED, Gambling, and Sleep Bruxism groups showed mechanical TW severity comparable to controls. Although these populations are associated with stress, anxiety, and bruxism, the bruxism itself tends to be intermittent and when it occurs predominantly in a centric pattern, the limited duration of tooth contact may reduce clinically detectable mechanical wear [10,65,66]. The predominance of chemical wear in the sleep bruxism group supports earlier findings that erosion may amplify occlusal TW [67]. Differentiating mechanical and chemical components remains challenging given the lack of standardized diagnostic criteria and limited clinical validation of wear patterns [43].

The results showed a strong association between the severity of chemical and mechanical TW per tooth, especially in high-risk groups, consistent with previous studies. Rius-Bonet et al. [29] found erosion increased attrition risk 6.3-fold in healthy young adults, highlighting the importance of preventive strategies targeting both processes. Chemical and mechanical factors act synergistically, as acid-induced softening enhances susceptibility to mechanical forces [27,67]. Clinically, this underscores the need to minimize acid exposure (e.g., diet, reflux, ED, salivary dysfunction) while controlling mechanical risks (e.g., bruxism, parafunctions, brushing technique).

Analysis of the salivary parameters revealed physiologically expected interrelationships. Physiologically, salivary flow rate and pH are inversely related to viscosity, as increased flow enhances bicarbonate secretion, elevates pH, and reduces mucin concentration, thereby decreasing viscosity. In contrast, buffering capacity primarily depends on the proportion of bicarbonates, phosphates, and specific proteins, and does not vary in direct proportion to flow rate or viscosity [27,68].

Chemical TW severity increased with age and BMI and decreased with the number of remaining teeth. Age-related cumulative acid exposure and reduced salivary protection, along with concentrated occlusal loads from tooth loss, increase susceptibility [10,27,69]. Higher BMI, often associated with GERD and acidic dietary patterns, also contributes to wear [17]. However, no association with acidic diet was observed, likely due to underreporting, cross-sectional design, or low exposure levels [14,70].

The severity of mechanical TW was positively associated with age, male sex, brushing force, and bruxism, and negatively with the number of teeth and frequency of toothbrushing. Aging increases cumulative exposure to masticatory forces and oral habits, while tooth loss concentrates functional load on fewer occlusal surfaces, amplifying attrition [10,71]. Literature has shown that inadequate brushing technique and excessive brushing force are associated with cervical abrasion [67,72]. However, there is no evidence that lower brushing frequency contributes to greater TW through improper technique or excessive force. Finally, bruxism, particularly involving eccentric tooth contact, has been linked to increased mechanical wear severity due to repetitive occlusal loading [10,73]. However, most evidence is cross-sectional, underscoring the need for longitudinal studies using standardized criteria for bruxism and TW assessment [19].

Most studies on TW do not differentiate chemical from mechanical processes. In contrast, our study separated these components and found no sex-related differences in chemical wear, but significantly greater mechanical wear in men, associated with higher maximum bite force and BMI. These results support previous evidence linking greater occlusal load from higher bite force in men to increased mechanical TW [23,74,75].

In the present study, age was positively associated with BMI, acidic dietary habits, SB, salivary dehydration, and increased viscosity, and while it was negatively associated with the number of teeth, perceived stress, salivary pH, and flow rate. Trends in overweight status, behavioural factors, and age-related physiological changes in salivary function may contribute to an elevated risk of TW in older adults [10,21,76]. Younger adults, despite higher stress, generally have more efficient salivary function [29,77].

Multivariate analysis adjusted for age and sex identified at-risk group membership as directly associated with chemical TW, with salivary pH mediating this relationship. Studies incorporating salivary parameters confirm that not all components have equal weight as risk factors. Madariaga et al. [21] reported that stimulated salivary pH had the strongest negative association with wear severity after adjusting for age, sex, and risk group, consistent with Nobre et al. [28], who identified reduced pH as a key predictor of dental erosion. Likewise, Mulic et al. [78] found risk group membership, such as GERD, to be a significant determinant of dental erosion. Collectively, these findings suggest that salivary pH may be a potential indicator of TW risk. The mediation model assessing maximum bite force as a link between age and mechanical TW showed both age and bite force positively associated with wear per tooth, even after adjusting for sex. However, no significant indirect effect of age through bite force was found. These findings are consistent with previous evidence identifying age as a marker of cumulative exposure and bite force as an indicator of occlusal load intensity, both exerting separate effects on structural dental loss [69,75]. It should be emphasized that the absence of bite force data for some participants at random could influence the interpretation of the mediation model; however, this study is designed to explore variable associations rather than to draw causal inferences.

Certain limitations in this study must be acknowledged. Technical and logistical challenges—such as strict eligibility criteria, limited participant availability, and extended recruitment timelines—prevented attainment of the planned sample size, especially for the TCA and ED groups. Consequently, statistical power may be reduced, and findings in these groups should be viewed as preliminary. The cross-sectional design precludes temporal analysis and limits causal inference. High variability among available TW assessment tools and methodological inconsistencies in salivary testing pose challenges for comparisons across studies and interpretation of findings. Although chair-side saliva test kits are convenient and user-friendly, their reliance on colorimetric pH and buffering evaluation provides only semi-quantitative results. Readings may fluctuate based on operator interpretation, which could affect precision. Additionally, reliance on self-reported data, potential residual confounding beyond age and sex, and recruitment from a tertiary care setting may affect external validity. Nevertheless, key strengths include rigorous group classification with standardized criteria, inclusion of a healthy control group, and novel data on GD as possible emerging at-risk profile. Exploratory modelling, along with efforts to separately assess chemical and mechanical wear, contributed to a better distinction of possible etiological factors. Understanding the predominant type of wear in each risk group supports the development of more targeted preventive and therapeutic strategies.

## 5. Conclusions

Belonging to an at-risk group, particularly individuals with GERD or ED, and reduced salivary pH were the variables most strongly associated with chemical TW. The effect of belonging to an at- risk group on chemical TW severity is influenced both directly and indirectly through changes in salivary pH. Patients diagnosed with GERD exhibited the highest severity of both chemical and mechanical wear, suggesting a synergistic interaction between acid erosion and attrition. Age and bite force were both positively associated with mechanical TW severity. The findings highlight the need for comprehensive prevention strategies that address both chemical and mechanical factors. Emphasizing the need for early identification, routine dental wear screening—including thorough patient history and salivary analysis—should be prioritized to identify high-risk populations. Such proactive approaches would support targeted interventions for controlling acid exposure and optimizing salivary function, ultimately improving preventive care and clinical outcomes. Further longitudinal studies are needed, employing standardized methodologies and criteria, as well as larger sample sizes, to track wear progression, clarify causal pathways involving chemical, mechanical, and salivary factors, and explore emerging risk profiles such as GD, thereby opening new avenues to investigate psychosocial and behavioral contributions to TW.

## Figures and Tables

**Table 1:** Comparison between control and risk groups in socio-demographical variables, general health and behavioral factors, and oral hygiene.

Characteristic	Total	Control	Risk	Cohen’s |d|/|h|	*p*-Value ^2^
	N = 147 ^1^	N = 45 ^1^	N = 102 ^1^		
**Socio-demographics**					
Age (years)	40 (15)	37 (12)	42 (16)	0.34	0.057
Sex				0.03	0.9
Female	96 (65%)	29 (64%)	67 (66%)		
Male	51 (35%)	16 (36%)	35 (34%)		
**General Health and Behavioural Factors**					
Natural teeth	27.1 (3.6)	28.1 (2.4)	26.6 (4.0)	0.41	0.11
Total teeth	27.89 (2.41)	28.36 (2.07)	27.69 (2.53)	0.28	0.2
BMI	25.3 (4.9)	24.2 (4.8)	25.8 (5.0)	0.31	**0.048**
Maximum Bite Force (N)	639 (208)	598 (201)	672 (210)	0.36	0.11
Duration of condition (months)	-	-	143 (139)	**-**	**-**
Medication				**0.81**	**<0.001**
No	63 (43%)	31 (69%)	32 (31%)		
Yes	84 (57%)	14 (31%)	70 (69%)		
Alcoholism				0.2	0.2
No	128 (87%)	37 (82%)	91 (89%)		
Yes	19 (13%)	8 (18%)	11 (11%)		
Tobacco				0.42	**0.029**
No	114 (78%)	40 (89%)	74 (73%)		
Yes	33 (22%)	5 (11%)	28 (27%)		
Drugs				0.38	0.10
No	140 (95%)	45 (100%)	95 (93%)		
Yes	7 (4.8%)	0 (0%)	7 (6.9%)		
Sport practicing				0.31	0.082
No	105 (72%)	28 (62%)	77 (76%)		
Yes	41 (28%)	17 (38%)	24 (24%)		
Swimming				0.24	0.2
No	131 (90%)	38 (84%)	93 (92%)		
Yes	15 (10%)	7 (16%)	8 (7.9%)		
**Oral Hygiene**					
Frequency of toothbrushing (num/day)	2.33 (0.79)	2.63 (0.53)	2.20 (0.85)	0.56	**0.003**
Powered toothbrushes				0.38	0.13
Manual	99 (69%)	36 (80%)	63 (64%)		
Electric	28 (19%)	5 (11%)	23 (23%)		
Both	17 (12%)	4 (8.9%)	23 (23%)13 (13%)		
Hardness of toothbrush				0.59	**0.018**
Soft	37 (28%)	17 (40%)	20 (23%)		
Medium	77 (59%)	25 (58%)	52 (60%)		
Hard	16 (12%)	1 (2.3%)	15 (17%)		
Force while toothbrushing (0–10)	5.98 (1.69)	5.40 (1.57)	6.24 (1.68)	0.51	**0.020**
Eating/drinking habits					
Acidic Diet (0–16)	4.63 (2.96)	3.83 (2.14)	4.98 (3.20)	0.39	0.13
**Bruxism/Oral Habits/Stress**					
Sleep Bruxism	0.88 (0.95)	0.33 (0.56)	1.13 (0.98)	**0.91**	**<0.001**
Awake Bruxism	0.65 (0.79)	0.27 (0.58)	0.81 (0.81)	**0.73**	**<0.001**
OBC2	26 (9)	24 (6)	27 (9)	0.26	0.2
PSS14	25 (9)	20 (8)	27 (9)	**0.77**	**<0.001**
**Salivary Characteristics**					
Increasing salivary viscosity				**0.98**	**<0.001**
Normal	89 (61%)	40 (89%)	49 (48%)		
Altered	58 (39%)	5 (11%)	53 (52%)		
Resting salivary pH	6.92 (0.58)	7.21 (0.46)	6.79 (0.59)	**0.77**	**<0.001**
Stimulated salivary flow (mL)	7.98 (3.18)	8.79 (3.27)	7.62 (3.09)	0.37	**0.031**
Buffering ability (0–12)	10.20 (2.52)	10.44 (2.41)	10.09 (2.57)	0.14	0.4
Unstimulated salivary flow (g)	2.95 (2.10)	3.18 (2.13)	2.86 (2.08)	0.15	0.2
**Severity of TW**					
Severity of chemical TW	22 (21)	5 (9)	29 (20)	**1.36**	**<0.001**
Severity of mechanical TW	18 (11)	14 (9)	20 (11)	0.56	**0.003**
Severity of chemical TW per tooth	0.86 (0.83)	0.19 (0.33)	1.15 (0.82)	**1.35**	**<0.001**
Severity of mechanical TW per tooth	0.68 (0.46)	0.49 (0.32)	0.77 (0.49)	0.62	**<0.001**

^1^ For continuous variables: Mean (SD). For categorical variables: n (%). ^2^ For continuous variables: Mann–Whitney U test. For categorical variables: Pearson’s Chi-squared test or Fisher’s exact test. Bold: *p*-value < 0.05; Cohen’s |d|/|h| > 0.7 (large effect). Abbreviations: BMI (Body Mass Index), OBC (Oral Behavior Checklist), PSS (Perceived Stress Scale), TW (Tooth Wear).

**Table 2:** Comparison between control and all different risk groups in socio-demographical variables, general health and behavioral factors, and oral hygiene.

Characteristic	Control	Bruxism	GERD	Gambling	EDs
	N = 45 ^1,2^	N = 28 ^1,2^	N = 34 ^1,2^	N = 20 ^1,2^	N = 20 ^1,2^
**Socio-demographics**					
Age (years)	37 (12) **C**	38 (14) **C,E**	54 (12) **A,B,D,E**	40 (14) **C,E**	28 (10) **B,C,D**
Sex	** D**	** D**	** D**	**A,B,C,E**	** D**
Female	29 (64%)	21 (75%)	23 (68%)	4 (20%)	19 (95%)
Male	16 (36%)	7 (25%)	11 (32%)	16 (80%)	1 (5.0%)
**General Health and Behavioural Factors**					
Natural teeth	28.1 (2.4) **C**	27.4 (1.8) **C**	24.8 (4.7) **A,B,E**	26.7 (4.5)	28.6 (3.0) **C**
Total teeth	28.36 (2.07)	27.64 (1.70)	26.94 (2.71) **E**	27.55 (3.24)	29.15 (1.81) **C**
BMI	24.2 (4.8) **C,E**	24.6 (4.0) **C,E**	28.9 (3.9) **A,B,E**	26.8 (5.2) **E**	21.1 (3.0) **A,B,C,D**
Maximum Bite Force (N)	598 (201)	762 (207)	661 (172)	547 (216)	692 (250)
Duration of condition (months)	-	129 (151)	131 (125)	199 (149)	128 (132)
Medication	**C**	**C**	** A,B,D,E**	**C**	**C**
No	31 (69%)	14 (50%)	1 (2.9%)	8 (40%)	9 (45%)
Yes	14 (31%)	14 (50%)	33 (97%)	12 (60%)	11 (55%)
Alcoholism					
No	37 (82%)	25 (89%)	31 (91%)	16 (80%)	19 (95%)
Yes	8 (18%)	3 (11%)	3 (8.8%)	4 (20%)	1 (5.0%)
Tobacco	** D**			** A**	
No	40 (89%)	23 (82%)	26 (76%)	10 (50%)	15 (75%)
Yes	5 (11%)	5 (18%)	8 (24%)	10 (50%)	5 (25%)
Drugs	** D**			** A**	
No	45 (100%)	27 (96%)	33 (97%)	15 (75%)	20 (100%)
Yes	0 (0%)	1 (3.6%)	1 (2.9%)	5 (25%)	0 (0%)
Sport practicing			** E**		**C**
No	28 (62%)	23 (82%)	29 (88%)	15 (75%)	10 (50%)
Yes	17 (38%)	5 (18%)	4 (12%)	5 (25%)	10 (50%)
Swimming					
No	38 (84%)	23 (85%)	32 (94%)	19 (95%)	19 (95%)
Yes	7 (16%)	4 (15%)	2 (5.9%)	1 (5.0%)	1 (5.0%)
**Oral Hygiene**					
Frequency of toothbrushing (num/day)	2.63 (0.53) **C,D**	2.66 (0.61) **C,D**	1.91 (0.77) **A,B,E**	1.78 (1.08) **A,D,E**	2.48 (0.64) **C,D**
Powered toothbrushes					
Manual	36 (80%)	18 (67%)	15 (45%)	16 (80%)	14 (74%)
Electric	5 (11%)	6 (22%)	12 (36%)	1 (5.0%)	4 (21%)
Both	4 (8.9%)	3 (11%)	6 (18%)	3 (15%)	1 (5.3%)
Hardness of toothbrush					
Soft	17 (40%)	7 (28%)	6 (21%)	5 (29%)	2 (13%)
Medium	25 (58%)	15 (60%)	18 (62%)	8 (47%)	11 (69%)
Hard	1 (2.3%)	3 (12%)	5 (17%)	4 (24%)	3 (19%)
Force while toothbrushing (0–10)	5.40 (1.57) **D**	5.66 (1.71)	6.28 (1.68)	6.93 (1.72) **A**	6.30 (1.45)
Acidic Diet (0–16)	3.83 (2.14)	4.11 (3.01)	5.53 (3.22)	5.50 (3.70)	4.75 (2.80)
**Bruxism/Oral Habits/Stress**					
Sleep Bruxism	0.33 (0.56) **B,C**	2.04 (0.67) **A,C,D,E**	0.88 (0.92) **A,B**	0.80 (0.77) **B**	0.60 (0.84) **B**
Awake Bruxism	0.27 (0.58) **B**	1.27 (0.70) **A,C,D,E**	0.69 (0.82) **B**	0.58 (0.78) **B**	0.63 (0.78) **B**
OBC2	24 (6)	29 (9) **D**	26 (10)	21 (9); **B,E**	30 (6) **D**
PSS14	20 (8) **D,E**	23 (7) **D,E**	23 (9) **D,E**	31 (6) **A,B,C**	34 (8) **A,B,C**
**Salivary Characteristics**					
Increasing salivary viscosity	**C,E**		** A**		** A**
Normal	40 (89%)	18 (64%)	10 (29%)	11 (55%)	10 (50%)
Altered	5 (11%)	10 (36%)	24 (71%)	9 (45%)	10 (50%)
Resting salivary pH	7.21 (0.46) **C,D,E**	7.10 (0.56) **C,E**	6.63 (0.61) **A,B**	6.80 (0.51) **A**	6.62 (0.52) **A,B**
Stimulated salivary flow (mL)	8.79 (3.27)	8.00 (3.39)	6.76 (2.99)	7.42 (2.62)	8.74 (3.02)
Buffering ability (0–12)	10.44 (2.41)	10.75 (2.24)	9.79 (2.76)	10.47 (1.74)	9.30 (3.16)
Unstimulated salivary flow (g)	3.18 (2.13)	3.50 (2.72)	2.56 (1.59)	3.13 (2.47)	2.17 (0.89)
**Severity of TW**					
Severity of chemical TW	5 (9) **B,C,D,E**	25 (21) **A,C**	37 (14) **A,B**	29 (24) **A**	24 (21) **A**
Severity of mechanical TW	14 (9) **C**	18 (9) **C**	26 (12) **A,B,D,E**	18 (10) **C**	14 (9) **C**
Severity of chemical TW per tooth	0.19 (0.33) **B,C,D,E**	0.91 (0.78) **A,C**	1.51 (0.58) **A,B,E**	1.17 (1.08) **A**	0.86 (0.75) **A,C**
Severity of mechanical TW per tooth	0.49 (0.32) **C**	0.65 (0.30) **C**	1.08 (0.58) **A,B,D,E**	0.68 (0.37) **C**	0.48 (0.31) **C**

^1^ For continuous variables: Mean (SD). For categorical variables: n (%). ^2^ For continuous variables: Mann–Whitney U test. For categorical variables: Pearson’s Chi-squared test or Fisher’s exact test. Adjusted using Holm method. Abbreviations: GERD (Gastroesophageal Reflux Disease), ED (Eating Disorder), BMI (Body Mass Index), OBC (Oral Behavior Checklist), PSS (Perceived Stress Scale), TW (Tooth Wear). To indicate statistically significant differences between particular groups, the following code is being used: Control (**A**), Bruxism (**B**), GERD (**C**), Gambling (**D**), ED (**E**). Each letter points out which groups are significantly different with the one the column is showing.

**Table 3:** Multiple regression model summary for chemical TW per tooth.

Predictors	Std. Coefficient	C.I. (95%)	*p*-Value
Group: Control	Reference		
Group: Sleep brux.	0.41	0.22–0.60	**<0.001**
Group: GERD	0.68	0.48–0.87	**<0.001**
Group: Gambling	0.39	0.18–0.61	**<0.001**
Group: EDs	0.32	0.09–0.54	**<0.001**
Salivary pH	−0.16	−0.29–−0.04	**<0.020**

Observations: 147; R2/Ra2: 0.469/0.450; Bold: Significant comparison (*p* < 0.05). Abbreviations: Std. (Standardized), C.I. (Confidence Interval), TW (Tooth Wear), GERD (Gastroesophageal Reflux Disease), ED (Eating Disorder).

**Table 4:** Multiple regression model summary for the mechanical TW per tooth model.

Predictors	Std. Coefficient	C.I. (95%)	*p*-Value
Maximum bite force	0.21	0.21–0.21	**0.008**
Age	0.74	0.73–0.74	**<0.001**

Observations: 84; R2/Ra2: 0.538/0.527; Bold: Significant comparison (*p* < 0.05). Abbreviations: Std. (Standardized), C.I. (Confidence Interval), TW (Tooth Wear).

**Table 5:** Mediation model summary of square root chemical TW severity per tooth in risk groups, mediated by salivary pH.

pH						
Predictor	Std. Coefficient	SE	*p*-Value	Std. Coefficient	SE	*p*-Value
Risk Group	−0.307	0.072	**<0.001**	0.534	0.053	**<0.001**
Salivary pH				−0.154	0.065	**0.018**
Sex	0.002	0.076	>0.9	0.068	0.06	0.258
Age	−0.184	0.075	**0.015**	0.216	0.06	**<0.001**
Fitness	R^2^ = 0.146			R^2^ = 0.466		

Bold: Significant comparison (*p* < 0.05). Abbreviations: TW (Tooth Wear), Std. (Standardized), SE (Standard error).

**Table 6:** Mediation model summary of square root mechanical TW severity per tooth by Age, mediated by Bite force.

Bite Force						
Predictor	Std. Coefficient	SE	*p*-Value	Std. Coefficient	SE	*p*-Value
Bite Force				0.173	0.08	**0.030**
Sex	0.367	0.09	**<0.001**	0.087	0.079	0.271
Age	−0.132	0.099	0.183	0.736	0.045	**<0.001**
Fitness	R^2^ = 0.156			R^2^ = 0.545		

Bold: Significant comparison (*p* < 0.05). Abbreviations: TW (Tooth Wear), Std. (Standardized), SE (Standard error).

## Data Availability

Individual-level data cannot be shared publicly because of participant privacy and ethics restrictions. Summary tables and the analysis plan/code are available from the corresponding author upon reasonable request and after institutional approvals.

## Abbreviations

The following abbreviations are used in this manuscript:

TW	Tooth wear
GERD	Gastroesophageal reflux disease
SB	Sleep bruxism
AB	Awake bruxism
ED	Eating disorders
GD	Gambling disorders
PSS	Perceived Stress Scale
OBC	Oral Behavior Checklist
TWES	Tooth Wear Evaluation System
BMI	Body mass index
